# Photoregulation of the biosynthetic activity of fungus *Inonotus obliquus* using colloidal solutions of biogenic metal nanoparticles and low-intensity laser radiation

**DOI:** 10.1080/21655979.2025.2458371

**Published:** 2025-01-28

**Authors:** Oksana Mykchaylova, Anatoliy Negriyko, Nadiia Matvieieva, Kostiantyn Lopatko, Natalia Poyedinok

**Affiliations:** a Department of Translational Medical Bioengineering, National Technical University of Ukraine “Igor Sikorsky Kyiv Polytechnic Institute”, Kyiv, Ukraine; b M.G. Kholodny Institute of Botany, National Academy of Sciences of Ukraine, Kyiv, Ukraine; c Department of Laser Spectroscopy, Institute of Physics of the National Academy of Sciences of Ukraine, Kyiv, Ukraine; d Institute of Cell Biology and Genetic Engineering, National Academy of Science of Ukraine, Kyiv, Ukraine; e National Academy of Science of Ukraine, National University of Life and Environmental Sciences, Kyiv, Ukraine

**Keywords:** *Inonotus obliquus*, colloidal solutions, laser, mycelial biomass, polysaccharides, flavonoids, melanin, *in vitro*

## Abstract

This article presents new data on the integrated use of colloidal solutions of nanoparticles and low-intensity laser radiation on the biosynthetic activity of the medicinal mushroom *Inonotus obliquus in vitro*. Traditional mycological methods, colloidal solutions of biogenic metals, and unique photobiological methods have also been used. It was found that colloidal solutions of nanoparticles of all metals used increased the growth characteristics of *I. obliquus* (55–60%), while irradiation of the fungal inoculum with laser light in a medium with nanoparticles reduced the growth activity of *I. obliquus* mycelia by 12.3–35.4%. Silver nanoparticles (AgNPs) in a nutrient medium suppressed the biosynthesis of extracellular polysaccharides, whereas laser irradiation in the same medium increased the synthesis of intracellular polysaccharides by 9.7 times. Magnesium nanoparticles (MgNPs) and iron nanoparticles (FeNPs) inhibited the synthesis of intracellular polysaccharides in the mycelial mass of *I. obliquus*. At the same time, laser irradiation of the inoculum with MgNPs, on the contrary, induced a sharp increase in the amount of polysaccharides in the culture liquid (20 times). Treatment of the inoculum in a medium with nanoparticles with a laser caused an intensification of the synthesis of flavonoids in the mycelial mass and an increase in the synthesis of melanin pigments (25–140%). The results obtained suggest the possibility of the complex use of colloidal solutions of Fe, Ag, and Mg nanoparticles and low-intensity laser radiation as environmentally friendly factors for regulating biosynthetic activity in the biotechnology of cultivating the valuable medicinal mushroom *I. obliquus*.

## GRAPHICAL ABSTRACT

**Figure:**
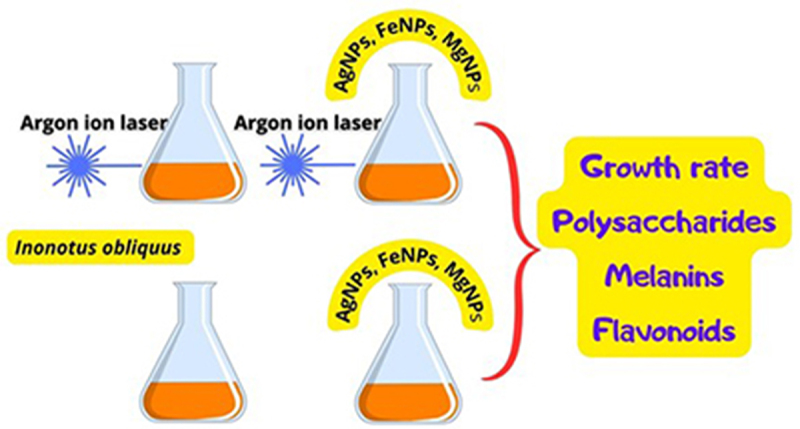

## 1. Introduction

Innovative achievements in nanotechnology have led to their successful application in biology, genetic engineering, medicine, pharmaceutical industries, and agro-industrial complexes possible [1–4]. Areas such as nanobiotechnology and nanomedicine have emerged based on modern nanotechnology. The small size of nanoparticles (NPs) affects their interactions with living organisms, including fungi. Biogenic metal NPs often have properties different from those of larger forms of the same metal, which makes them useful for a variety of applications, including industrial uses [5,6]. It is important to understand how nanoparticles interact with biological entities to safely apply nanotechnology in modern biology, and medicine.

One of the unique features of nanoparticles is their ability to penetrate cells and interact with intracellular elements while maintaining their shape and properties [7]. The influence of various materials as biological agents in the form of nanoparticles on the development of living organisms has been actively studied in recent years, including in mycology at the stages from the germination of fungal spores to the formation of fruiting bodies [8–10]. However, given the wide range of biochemical processes in fungal organisms, there remains a wide range of insufficiently studied issues regarding the influence of nanoparticles on the development of fungi, requiring additional detailed studies. Among these insufficiently studied phenomena are the combined effects of biogenic metal nanoparticles and low-intensity artificial irradiation on fungal processes. Both factors (nanoparticles and artificial lighting, in particular, laser) independently demonstrate the ability to stimulate and inhibit the development of living organisms, particularly plants [11–13]. The synergistic action of two independent factors – metal nanoparticles and lasers – can open up new effective mechanisms for controlling processes in biological systems, mainly through photocatalysis and enhanced photostimulation of processes in the presence of nanoparticles.

Previous studies have demonstrated the effect of colloidal solutions of metal nanoparticles AgNPs, FeNPs, and MgNPs on the biosynthetic activity of the medicinal mushroom *Inonotus obliquus* (Ach.: Pers.) Pilát [14]. However, the study of the simultaneous influence of colloidal solutions of metal nanoparticles and artificial lighting on the cultivation of macromycetes *in vitro* has not been conducted.

Experimental studies on the combined influence of laser light and colloidal solutions of nanoparticles, using the valuable medicinal macromycete *I. obliquus* as a model object, will provide new data on the growth characteristics and biosynthetic activity of the fungus when exposed to photoactivated nanoparticles.

The pharmacological properties of the medicinal mushroom *I. obliquus*, known in medical practice as ‘chaga,’ are determined by the biological activity of the complex of various compounds that make up its composition. More than 250 bioactive compounds have been isolated and identified from the fruiting bodies and mycelia of *I. obliquus*, including polysaccharides (β-glucans and heteroglucans), polyphenol derivatives, melanins, betulin, lanostane-type triterpenoids [15,16]. Among all the biologically active compounds of the fruiting bodies and mycelial mass of *I. obliquus*, it is polysaccharides that are of interest due to a wide range of pharmacological properties, such as antioxidant, anticancer, hypoglycemic, anti-inflammatory [17–19]. Polysaccharides of *I. obliquus* act as biological response modifiers, stimulate the immune system, and possess a wide range of immunopharmacological activities [20]. Clinical studies have shown that chaga polysaccharides are responsible for modulating humoral and cellular mediators such as interleukins, macrophage activators, T-helpers, and natural killers (NK). They can stimulate the release of various cytokines (IL-1α, IL-2, IFNγ, TNFα) and may be involved in combination therapy for cancer treatment [20,21]. In addition, the polysaccharides include monomers that participate in the antioxidant process, stimulating the increased production of free antioxidant enzymes, reducing the number of radicals in the body, and thereby contributing to lipid oxidation and regulating lipid metabolism [22]. It has been confirmed that phenols and flavonoids of aqueous-alcoholic extracts of *I. obliquus* exhibit strong antioxidant activity [23,24]. The therapeutic activity of *I. obliquus* extracts is largely provided by the main component – fungal melanin, the content of which is about 50–60% of the extractive substances [17,18]. According to Lindequist (2024), the presence of a wide range of metabolites in medicinal mushrooms allows considering mushroom raw materials and extracts from them as complex multi-component mixtures. Unlike the action of a single substance, multi-component mushroom mixtures exhibit a broad spectrum of action, thus being regarded as multi-purpose medicinal products [25,26]. Owing to their complex action, mushroom raw materials and chaga extracts have shown significant antioxidant, antimicrobial, and immunomodulatory activities [27]. In addition, aqueous chaga extract is a powerful immunomodulator that restores the bone marrow system damaged by chemotherapy [27].

Taking into account the above, the purpose of our work was to study the influence of colloidal solutions of biogenic metals on the growth characteristics and biosynthetic activity of the medicinal basidiomycete *Inonotus obliquus*, as well as the effects of photocatalytic activity of NPs after exposure to low-intensity laser irradiation under submerged cultivation conditions.

## 2. Materials and methods

### 2.1. Mushroom sample

A pure axenic culture of the basidiomycete of *Inonotus obliquus* was chosen as the model object. The strain of *I. obliquus* IBK 1877 was supplied by the Mushrooms Collection of the M.G. Kholodny Institute of Botany of the National Academy of Sciences of Ukraine (*IBK*) (https://www.gbif.org/dataset/148aefb6-cf88-443e-9780-b774ef7333e7) (Supplemental Material 1). The pure culture was maintained on the Malt Extract Agar (Thermo Fisher Scientific, USA) slants at 4°C.

### 2.2. Preparation of colloidal solutions of metal nanoparticles

In the experiment, aqueous colloidal solutions of nanoparticles of biogenic metals such as Fe, Mg, and Ag were used, obtained by electro-spark processing of conductive materials [28]. The method of electro-spark synthesis of metal nanoparticles (conductive materials) is based on the phenomenon of electrical erosion of the surface layers of metal granules, which is accompanied by the evaporation and condensation of metal vapors to form nanoparticles. This method allows for obtaining metal nanoparticles within a narrow distribution range, namely 20–50 nm. According to the generally accepted classification of morphology, the obtained nanoparticles mainly belong to 3-D crystals. We implemented a method for producing nanoparticles in a semi-industrial technological complex, which includes bit pulse generators, discharge chambers and a process control unit for electrical parameters (Supplemental Material 2).

The use of discharge pulse power generators (DPPG) for the synthesis of metal nanoparticles makes it possible to obtain low-temperature plasma in the discharge channel in a short period of time, which ensures effective erosion of the anode material. A unique feature of the volumetric electrospark dispersion method is the presence of a conductive layer of granules located between the main electrodes. The process takes place in a reaction chamber filled with a weakly conductive liquid, namely: deionized water.

**Figure 1.**
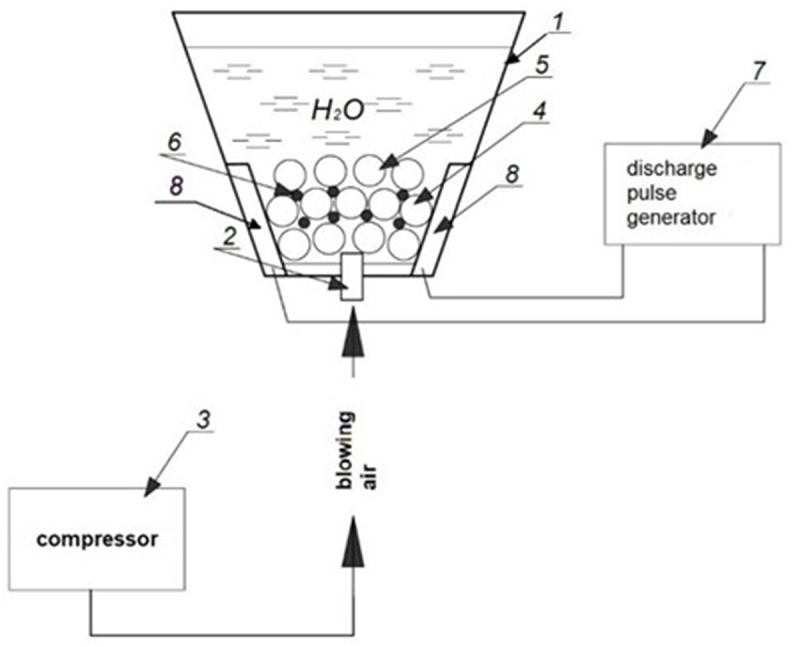
Technological complex for obtaining metal nanoparticles. 1 – discharge chamber; 2 – air sprayer; 3 – compressor; 4.5 – metal granules; 6 – discharge (plasma) channel; 7 – discharge pulse generator; 8 – electrodes (anode, cathode).

Voltage supply to the main electrodes are caused by the passage of current through a chain of freely stacked granules in the stochastic switching mode. The use of low voltages (up to 150 V) and small interelectrode gaps makes it possible to provide modes when up to 85% of all accumulated energy on the capacitor is used for local heating of the surface of the contacting granules. A layer of conductive metal granules with a maximum diameter of 5–10 mm is used to ensure the performance of spark erosion dispersion instead of a single contact gap. With the help of a thyristor generator with capacitive energy storage, discharge pulses were obtained, the amplitude of the voltage of which was regulated in the range of 40–150 *V*, and the current – from 30 to 500 *A* (Figure 1).

The system of metal granules and plasma channels immersed in a dielectric liquid and ohmic contacts between them is a complex electrical load. It has a significant nonlinear character and significant parametric dependencies on a number of technological factors: the frequency of discharge pulses, the flow rate and the temperature of the working fluid, etc. In addition, the physical nature of the occurrence of spark discharges in the layer of conductive granules determines the stochastic migration of plasma channels along their surface and switching current flow paths to adjacent pairs of granules. As a result, the instantaneous values of the equivalent electrical resistance of such a medium vary widely: from 0.05 to 100 *ohms*. The frequency of discharge pulses is regulated in the range of 100–200 *Hz* and the inductance of the discharge circuit *L* does not exceed 1 *μH*.

Nanoparticles were synthesized in discharge chambers filled with deionized water with low electrical conductivity. As a result of electric spark erosion of granules of the corresponding metals (iron, silver and magnesium), colloids of these metals are formed, the dispersed phases of which are metal nanoparticles. To obtain metal colloids, we used chemically pure starting materials (99.95% of the content of the corresponding metal) and deionized water with an electrical conductivity of no more than 20–30 microSiemens (μS). No other chemicals were present. The electrokinetic potential (zeta potential), sedimentation stability, and the size of the solid (dispersed) phase make it possible to use the resulting colloidal solutions in biology and biotechnology. The physiological component of the nanoparticles used in our experiment is due to the fact that they are an integral part of all physiological processes that occur in the fungal mycelium and perform both structural and catalytic functions. Colloidal solutions of nanometals were added to the glucose-peptone-yeast (GPY) nutrient medium at a concentration of 10^−10^ M. When choosing the concentration, we were guided by the results obtained by other researchers, as well as our data [14,29,30].

### 2.3. Analysis of nanoparticles using scanning electron microscope and transmission electron microscope

The structure, size distribution, and morphology of metal nanoparticles were investigated using scanning electron microscope (SEM) and transmission electron microscopy (TEM) methods. The particles’ size, shape, and chemical composition were determined using a scanning electron microscope MIRA3 TESCAN. After preparation (synthesis), the obtained metal nanoparticles were deposited onto a copper microgrid coated with an amorphous carbon film and dried in preparation for examination using transmission electron microscopy (TEM). Transmission electron microscopy was performed using Titan 80–300 and JEOL JEM-100SXII electron microscopes, operating at acceleration voltages of 100 kV and 300 kV, respectively.

### 2.4. X-ray structural analysis

The phase composition of nanoparticles was analyzed by X-ray diffraction on an Ultima-IV diffractometer (Rigaku, Japan) based on the angular dependences of the intensities of the diffraction peaks of scattered monochromatic radiation (k = 0.15418 nm) [31].

### 2.5. Determination ζ-potential of colloids

The value of the ζ-potential of colloids was determined using the Zetasizer Nano ZS analyzer using the reverse dispersion method (Malvern Instruments Ltd, United Kingdom).

### 2.6. Inoculum preparation and culture conditions

Cultivation of the inoculum of *I. obliquus* was axenically under controlled temperature (26°C), agitation (120 rpm), and dark for 12 days, carried out on a basic liquid nutrient medium and its modification (medium A – C) with the addition of a colloidal solution of metal nanoparticles. The basic medium (control) ‘glucose-peptone-y east’ (GPY) was composed per liter as follows: glucose (25.0 g), peptone (3.0 g), yeast extract (2.0 g), KH_2_PO_4_ (1.0 g), K_2_HPO_4_ (1.0 g), MgSO_4_ 7 h_2_O (0.25 g), pH 5.5 [18]. Modification with the addition of colloidal solution of metal nanoparticles:

medium A: GPY with silver nanoparticles (AgNPs);

medium B: GPY with iron nanoparticles (FeNPs);

medium C: GPY with magnesium nanoparticles (MgNPs).

100 mL of each liquid nutrient medium was placed in Erlenmeyer flasks with a capacity of 0.5 L, then sterilized in an autoclave for 0.5 h at a temperature of 120°C. After sterilization, inoculation of 10% of the volume was carried out and cultured under the conditions described earlier.

### 2.7. The influence of biogenic metal nanoparticles and laser radiation on growth characteristics and biosynthetic activity

**Figure 2.**
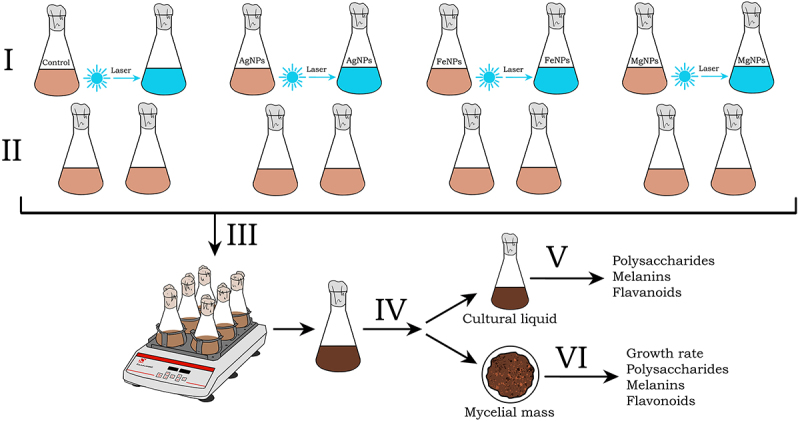
Scheme for studying the effect of metal nanoparticles and low-intensity laser irradiation on the growth characteristics and biosynthetic activity of *I. obliquus in vitro*: I – inoculum preparation (cultivation on a nutrient medium with colloidal solutions of nanometals and irradiation with low-intensity laser (488 nm); II – addition of inoculum *I. obliquus* into erlenmeyer flasks with a nutrient medium (GPY); III – cultivation under submerged culture conditions; IV – separation of the mycelial mass from the culture liquid; V – determination of the number of exopolysaccharides, flavonoids, melanins in the culture liquid; VI – determination of growth characteristics, content of intracellular polysaccharides, flavonoids, and melanins in the mycelial mass.

The influence of metal nanoparticles and low-intensity laser irradiation on the growth characteristics and biosynthetic activity of *I. obliquus in vitro* was studied using a method developed by the authors. The following scheme was used, as shown in Figure 2.

A pre-grown physiologically active inoculum of *I. obliquus* from each flask variant was used as a control (without irradiation), and the other part was irradiated with blue laser light (λ = 488 nm). After irradiation, the inoculum was added in an amount of 10% of the total volume into flat-bottomed Erlenmeyer flasks with a capacity of 0.5 L with 100 mL of GPY nutrient medium.

### 2.8. Irradiation

An experimental setup was created at the Institute of Physics of the National Academy of Sciences of Ukraine to conduct experimental studies on the impact of monochromatic laser irradiation on the biosynthetic activity of edible and medicinal mushrooms *in vitro*. The setup included a basic argon-ion laser LGN-106M1 and devices to measure laser output power (Thorlabs PM100D digital optical power and energy meter) and wavelength (USB2000 fiber optic spectrometer) (Supplemental Material 3).

The use of coherent (laser) radiation allowed the study of short-term effects of monochromatic light on the development of macromycetes, and it was established that the most significant effect was observed at doses of 240 mJ/cm^2^ [32–36]. To facilitate comparison with previous studies, regimens maintaining this dose were used in subsequent research [36,37]. This study used blue laser light with a wavelength of 488 nm and the same experimental conditions (irradiation dose, laser energy, energy density, exposure time, and irradiation geometry) as previous work [14,33–37]. These standardized approaches provided a basis for comparing the effects of low-intensity light on the growth and development of mushrooms *in vitro*. The exposure duration could be adjusted based on the power of the radiation source to achieve a dose of 240 mJ/cm^2^, allowing for flexibility in the duration of substrate irradiation, ranging from several tens to several hundred seconds. In our study, we irradiated the *I. obliquus* inoculum for 180 s.

Mushroom mycelium irradiation was carried out in flasks (the thickness of the layer of the nutrient medium with mycelium was no more than 1 cm). After irradiation, all experimental samples, irradiated and non-irradiated, were cultivated on an orbital shaker (120 rpm) at 25°C in the dark for 12 days. The mycelial biomass produced by each treatment was harvested by vacuum filtration to separate the culture broth. The fungal biomass was washed several times with distilled water and oven-dried in a TCO-80 MICROmed thermostat (Shanghai Youding International Yrade Co., LTD) at 60°C until constant weight.

### 2.9. Determination of intracellular polysaccharides

The content of intracellular polysaccharides (IPS) in the mycelial mass was determined according to the standard method described in Bisko et al. (2020). Briefly, a 1.0 g sample of the mycelial mass, dried to constant weight, was ground to a particle size of 0.1 ± 0.01 mm. To remove impurities of lipids and phenolic compounds from the mycelial mass, extraction was carried out with 96% ethyl alcohol in a ratio of 1:5 (weight : volume) with exposure for six h at a temperature of 24°C. The precipitate was filtered and IPS were extracted with a five-fold volume of bidistilled water for 20 h in a laboratory water bath WB-10 MICROMed (Shanghai Youding International Yrade Co., LTD) at a temperature of 80.0 ± 0.1°C. After this, the sediment was separated by centrifugation on a laboratory centrifuge MICROmed SM-3.01 (Shanghai Youding International Yrade Co., LTD) at 3,000 g for 20 min. The supernatant liquid was drained and precipitated with 96% ethyl alcohol in a ratio of 1:2 (volume). To ensure complete precipitation, the extract was left for 24 h at a temperature of 4.0 ± 0.1°C. The sediment was centrifuged at 3,000 g of liquid for 25 min and resuspended in hot double-distilled water (90 ± 0.1°C). The resulting IPS fraction was dried to a stable mass at a temperature of 60 ± 0.1°C in a TCO-80 MICROmed thermostat (Shanghai Youding International Yrade Co., LTD). The amount of IPS was determined by weighing (electronic analytical AS 62.R2 (Shanghai Youding International Yrade Co., LTD) and determined as a percentage of the dry weight of the mycelium [38].

### 2.10. Determination of extracellular polysaccharides

The amount of extracellular polysaccharides (EPS) in the culture liquid was determined using the weight method described in Mykchaylova et al. (2023). Briefly, for the determination of EPS, the obtained culture fluid after mycelia removal was concentrated in a vacuum evaporator (Unipan, Warszawa, Poland) three times from the initial volume, precipitated with 96% cooled ethanol in a ratio of 1:1 and placed in a refrigerator at 4°C for 24 h. The precipitate was isolated from the supernatant by centrifugation at 8,000 g for 15 min. After separation, EPS was dried at 60°С to constant weight. The yields of EPS were expressed as the g dry weight/L of the culture liquid [36].

### 2.11. Determination of flavonoides

Flavonoid content was studied spectrophotometrically by a slightly modified method [39] using spectrofluorimeter Panorama (λ = 510 nm). For this assay, the extract (0.25 mL) was mixed with deionized water (1 mL) and a 5% NaNO_2_ solution (0.075 mL). After 5 min, 0.075 mL of a 10% AlCl_3_ solution was added to the reaction mixture. NaOH (1 M, 0.5 mL) and deionized water (0.6 mL) were added to the final stage. The concentrations were calculated using a calibration curve y = 0.8574х (R^2^ = 0.9758) and were expressed as rutin equivalents (mg RE/g).

### 2.12. Determination of melanin

Isolation and identification of melanin in the mycelium of *I. obliquus* were carried out according to the standard methods described in Poyedinok et al. (2015). Dry mycelial mass was ground to a particle size of 0.1 ± 0.01 mm. Melanin extraction was carried out by alkaline hydrolysis using a 2 N NaOH solution in a 1:10 ratio, followed by precipitation with concentrated HCl. The coagulated pigment was separated by centrifugation at 10,000 g for 15 min. To remove carbohydrates and proteins, the resulting precipitate was purified by acid hydrolysis using 6 M HCl at a temperature of 100.0 ± 1°C for 2 h. To wash out lipids, the precipitate was treated with organic solvents (chloroform, ethyl acetate, and ethanol). The obtained melanin fraction was dried to constant weight at a temperature of 60.0 ± 1°C, and its quantity was determined by a gravimetric method. The yield of melanin synthesis was determined as the amount (mg) per unit volume of the nutrient medium (ml) over a certain cultivation period [34]. The experiments were conducted in triplicate. Melanin pigments were identified by qualitative reactions with KMnO4 (5%); oxidation with a 50% H_2_O_2_ solution, and by determining their solubility in organic and inorganic solvents.

### 2.13. Statistical analysis

The experiments were conducted in tri-replicates. Statistica software (version 6.0) was used for processing the results. The significance of the differences between the results was assessed using the Student’s t-test, and significance was accepted for *p* values < 0.05.

## 3. Results

### 3.1. Characteristics of metal nanoparticles

The impact of nanoparticles on biophysical and biochemical processes in living organisms is mainly determined by their chemical composition and interaction with the environment, which is associated with their small size. For metal nanoparticles, even a slight change in size by a few nanometers can significantly affect their optical properties and interaction with biological objects, such as cellular biomolecules. Due to the quantum confinement effect, nanoparticles acquire new optical and electromagnetic properties, which expands their scope of application. In addition, the morphology and size of nanoparticles play a key role in determining such characteristics as circulation time, biodistribution, and endocytosis mechanism. Therefore, the synthesis of nanostructures with a controlled homogeneous shape and size is extremely important for both fundamental research and practical applications. In this work, we also took into account an important conceptual provision – the choice of a preparative form or carrier for metal nanoparticles. The powder form, which is the most common in nanomaterial synthesis technologies, has a number of limitations for use in biotechnological purposes. Consolidation of dispersed phase particles leads to the loss of valuable properties characteristic of nanoscale materials. In addition, the formation of agglomerates makes it impossible to accurately dose the components when using minimal amounts of the solid phase, which is a serious limitation for the use of powder materials for biotechnological purposes. Therefore, one of the main requirements for such materials is to ensure a dispersed-isolated state of the solid phase. Taking these requirements into account, the most effective, in our opinion, is the method of electric spark dispersion in a liquid to obtain a colloidal form of a substance. The method of volumetric electric spark dispersion of metals in a liquid (deionized water) that we used allowed us to obtain colloidal solutions of AgNPs, FeNPs and MgNPs with sizes from 20 nm to 60 nm (Table 1). In addition, due to the sedimentation process natural for the colloidal form of a substance, the largest particles precipitate within a short time (up to 1 day). In this way, systems close to monodisperse are formed, which allows us to speak of a narrow distribution of solid phase particles. Technologies based on this method have advantages over technologies using the explosion of conductors, evaporation-condensation, mechanical, chemical, electron-beam and gas-thermal dispersion of materials. The average temperature of the working fluid may be low, but in microplasma volumes of sparks, ultra-high temperatures (7–10)×10^3^ K temporarily (for 10–100 μs) arise, which allows obtaining particles from materials with significantly different melting temperatures. High cooling rates of dispersed spark-erosion particles in liquid lead to significant modification of their structure and formation of materials with unique properties.

**Table 1.** Characteristics of colloidal solutions of nanoparticles obtained by electrospark processing of conductive materials.

Sample	Phase content		Size, nm
	Phase	Mass fraction, %	
AgNPs	Ag	100	30 – 50
FeNPs	α – Fe Fe_3_O_4_	60 – 7030 – 40	20 – 30
MgNPS	Mg MgO	30 – 5050 – 70	40 – 60

**Figure 3.**
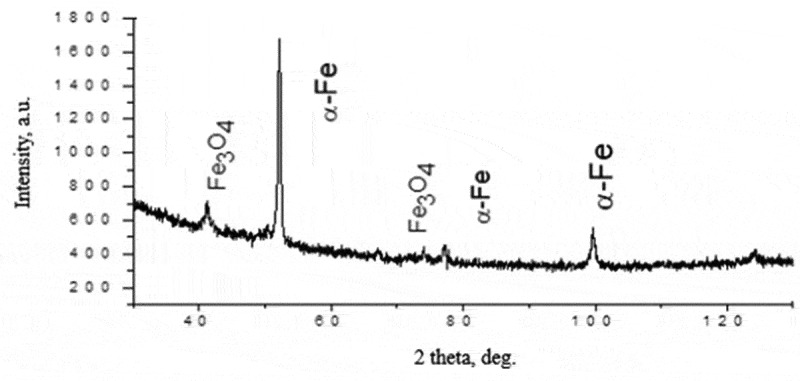
X-ray structural analysis of FeNPs.

**Figure 4.**
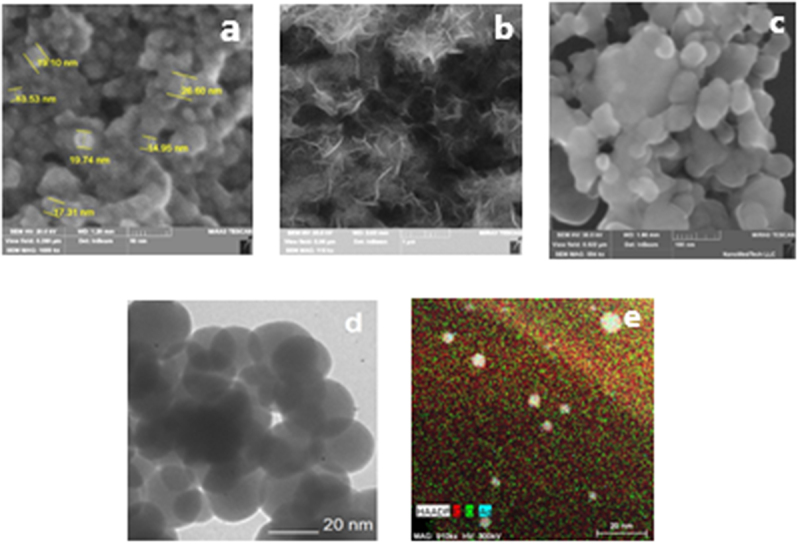
Micrograph of nanoparticles obtained by scanning electron microscopy (SEM): a – Fe, b – Mg, c – Ag. Micrograph of nanoparticles obtained by transmission electron microscopy (TEM): d – Fe, e – Ag.

**Figure 5.**
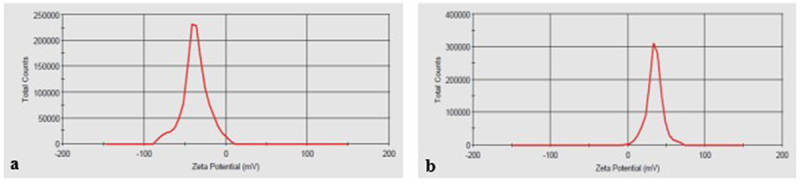
Value of ζ-potential on the surface of colloidal particles: a – Ag, b – Fe.

In our study, we utilized scanning electron microscopy (SEM), transmission electron microscopy (TEM), and X-ray structural analysis to analyze the morphological properties of nanoparticles. The properties of nanoparticles in the experimental colloidal solutions, obtained via spark erosion of granules of iron, silver, and magnesium, are detailed in Table 1 and Figures 3, 4, 5, respectively.

Based on the intensity of the diffraction pattern peaks, the colloidal solutions of iron and magnesium nanoparticles we studied consist of metals and metal oxides, with a mass fraction of up to 70% and nanoparticle sizes ranging from 20 nm (iron) to 60 nm (magnesium).

The SEM images of AgNPs, FeNPs, MgNPS nanoparticles obtained by electrospark processing of granules in liquid are shown in Figure 4. Analysis of the size and shape of nanoparticles showed that under the conditions of our experiment, nanoparticles were formed with a size from 20 to 60 nm and a coefficient shape (the ratio of the maximum size to the minimum) from 1 to 5. Iron nanoparticles were mainly globules with a shape factor from 1 to 2 and a size from 20 to 30 nm (Figure 4a). The size range of magnesium nanoparticles is 40–50 nm. A needlelike shape was predominantly observed (Figure 4b). For silver, a globular shape was characteristic (Figure 4c). The average particle size was 30–50 nm.

One of the most important parameters characterizing the aggregate stability of the colloidal system and its functionality is the electrokinetic potential (ζ-potential), which was measured using Malvern ZETA SIZER. Taking into account the conditions for obtaining an aqueous solution of metal nanoparticles (silver, iron, magnesium), deionized water is considered not only as a dispersion medium, but also as an active factor participating in the formation of the micellar structure of the dispersed phase. At the achieved plasma temperatures in the discharge channel of about 8 × 10^3^ K, water dissociates with the formation of atomic and molecular forms: O₂, H₂, O^−^, H^+^, OH^−^. The obtained results of measuring the ζ-potential (Figure 5) indicate the qualitative composition of the adsorption layer, which, together with the aggregate, forms a colloidal particle. The negative charge is formed due to the adsorption of oxygen atoms and a hydroxyl group. The ζ-potential value is about 30 mV, which is sufficient to ensure the aggregate stability of the system and to conduct further biological analyses and tests. The size of the nanoparticles and the ζ-potential value are of key importance for determining the transport of nanoparticles inside the cells of living organisms.

Analysis of the formation of the phase composition of metal nanoparticles indicates that a nanohydrate oxide film is formed on their surface, the thickness of which depends on the metal itself and the conditions of the electric spark treatment. This is a feature of the method, as well as the production of a colloidal form of metals when deionized water is used as a dispersion medium. The separation of particles by size is quite narrow, the maximum size does not exceed 100 nm, and they are almost completely chemically and electrically neutral. Preliminary certification of the obtained materials allows us to talk about the probable biological functionality of nanoparticles and the possibility of their use in biotechnology, in particular, in the technology of mushroom cultivation.

Thus, the electrokinetic potential (ζ-potential), sedimentation stability, and the size of the solid (dispersed) phase of the colloidal solutions of metal nanoparticles (AgNP, FeNP, MgNP) used by us suggest the possibility of their use for processing *I. obliquus* inoculum under the conditions of our experiment.

### 3.2. Effect of photoactivated colloidal solutions of metal nanoparticles on the growth activity

**Figure 6.**
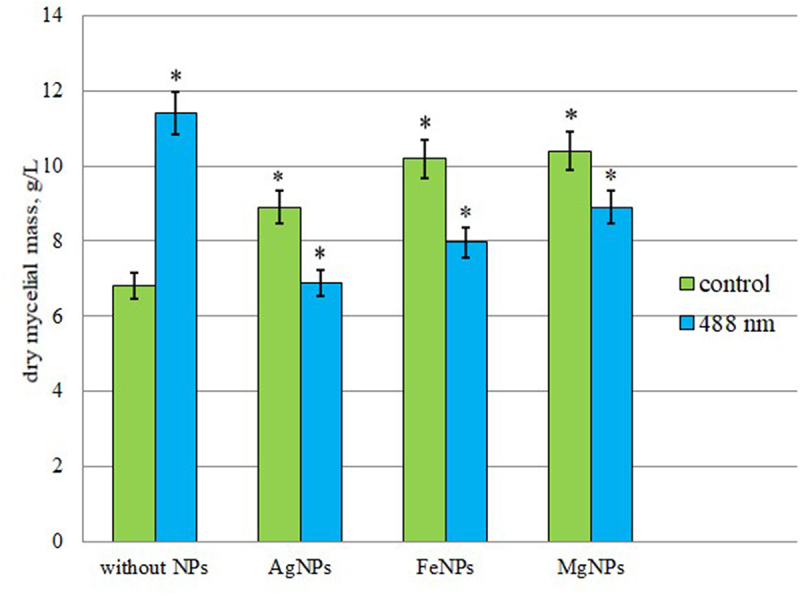
Growth activity of *Inonotus obliquus* after exposure to low-intensity laser radiation λ = 488 nm in a medium with nanoparticles. «*» indicates statistically significant differences in growth activity after irradiation in media with NPs (*p* < 0.05).

It was found that laser light irradiation (λ = 488 nm) of *I. obliquus* ІВК 1877 inoculum in a nutrient medium without nanoparticles led to an increase in the amount of mycelial mass from 11.3 ± 0.3 g/L to 13.9 ± 0.2 g/L, which was 23.1% higher than in the control (without irradiation and without nanoparticles) (Figure 6). The addition of colloidal solutions of nanoparticles used in the study (AgNPs, FeNPs, MgNPs) to the *I. obliquus* inoculum stimulated growth activity by 55–60% compared to the control (mycelium without nanoparticles). The greatest effect was observed for AgNPs and MgNPs – the accumulation of mycelial mass in these modes ranged from 17.5 ± 0.2 g/l (AgNPs) to 18.1 ± 0.3 g/l (MgNPs) on the twelfth day of cultivation and did not differ statistically. It should be noted that irradiation with laser light (λ = 488 nm) of the inoculum in a medium containing NPs reduced the growth activity of the mycelium induced by NPs in all experimental variants. The greatest effect of inhibition of growth activity was recorded for the medium with MgNPs – 35.4%, the least for FeNPs – 12.3% (Figure 6).

### 3.3. Effect of photoactivated colloidal solutions of metal nanoparticles on the synthesis of polysaccharides

**Figure 7.**
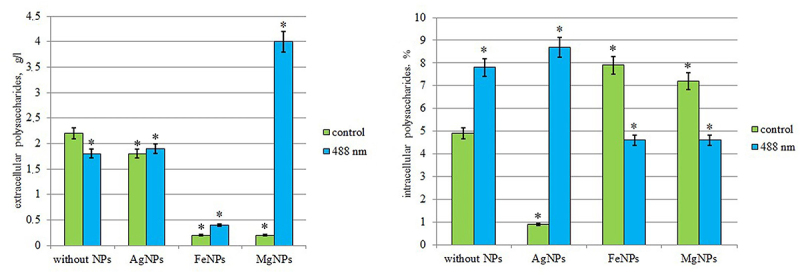
Synthesis of polysaccharides from *Inonotus obliquus* after exposure to low-intensity laser radiation λ = 488 nm in a medium with nanoparticles.

According to the results of our study, it was found that low-intensity laser radiation (λ = 488 nm) in a medium without nanoparticles induces the synthesis of intracellular polysaccharides (IPS) in the mycelial mass of *I. obliquus*, their amount increased by 59.1%. At the same time, under the same regime, a decrease in the amount of extracellular polysaccharides (EPS) in the culture fluid by 22.2% was recorded (Figure 7).

«*» – indicates statistically significant differences in the synthesis of polysaccharides after irradiation in media with NPs (*p* <0.05).

It was found that in the nutrient medium with AgNPs, a decrease in the biosynthesis of both EPS and IPS was observed compared to the control medium (without NPs) by 18.2% and 81.6%, respectively. At the same time, irradiation of the *I. obliquus* inoculum with low-intensity laser light in this medium significantly increased the amount of IPS by 9.7 times and statistically did not affect the synthesis of EPS.

In the nutrient medium with FeNPs, an increase in IPS synthesis by 1.6 times and a tenfold decrease in EPS synthesis were observed compared to the control medium (without NPs) (Figure 7). Irradiation of the *I. obliquus* inoculum in the medium with FeNPs inhibited the synthesis of IPS, whose amount decreased by 1.7 times, while a twofold increase in the concentration of EPS was recorded under this condition. Overall, the use of low-intensity laser light to treat the FeNPs inoculum is impractical for polysaccharide production.

In the nutrient medium with MgNPs, the IPS level increased by 46.9%, while the amount of EPS decreased by 91% compared to the control medium (without NPs). At the same time, irradiation of the *I. obliquus* inoculum with laser light in the medium with MgNPs caused a significant increase in the amount of EPS in the culture fluid (by 20 times) and reduced the amount of IPS by 36.2%

### 3.4. Effect of photoactivated colloidal solutions of metal nanoparticles on the synthesis of flavonoids

**Figure 8.**
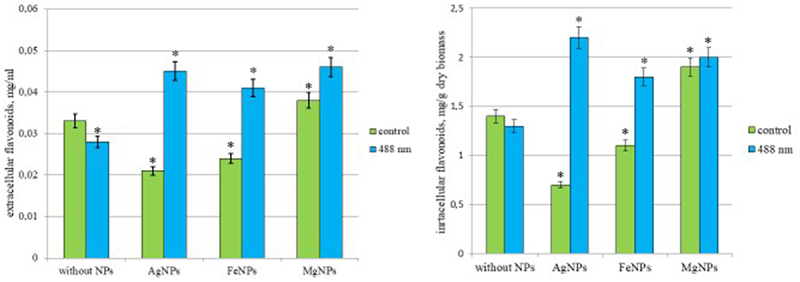
Synthesis of flavonoids *Inonotus obliquus* after exposure to low-intensity laser radiation λ = 488 nm in a medium with nanoparticles. *– statistically significant differences in the synthesis of flavonoids after irradiation in media with NPs are indicated (*p* < 0.05).

Analysis of the extracellular flavonoid content in the culture fluid showed their insignificant amount – the concentration did not exceed 0.03 mg/mL in the control medium (without nanoparticles), and the maximum amount was 0.045 mg/mL for media with silver and magnesium nanoparticles after irradiation (Figure 8). Therefore, under the conditions of our experiment, only intracellular flavonoids may be of interest. All photo-induced nanoparticles we used caused an intensification of the synthesis and accumulation of intracellular flavonoids in the mycelial mass of *I. obliquus* (Figure 8). The greatest effect of photostimulation on the accumulation of intracellular flavonoids was observed when using photoinduced AgNPs. In this variant, the flavonoid content was 3.14 times higher than in the control (without nanoparticles). The effects of stimulation of intracellular flavonoid synthesis by photoinduced MgNPs and FeNPs did not differ statistically, and their amount did not exceed 2.1 ± 0.3 mg/mL. The obtained data indicate the possibility of using only photoinduced AgNPs to intensify the synthesis of flavonoids in *I. obliquus*

### 3.5. Effect of photoactivated colloidal solutions of metal nanoparticles on the synthesis of melanin

**Figure 9.**
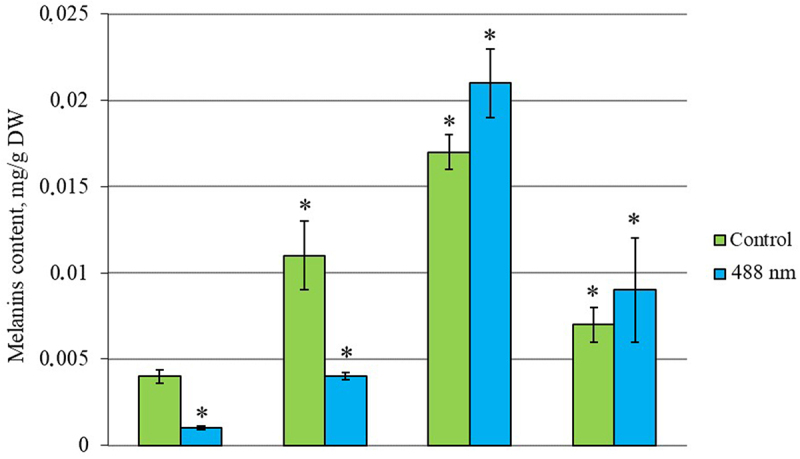
Synthesis of melanin by *Inonotus obliquus* after exposure to low-intensity laser irradiation λ = 488 nm in a medium with nanoparticles. *– indicates statistically significant differences in the synthesis of melanin after irradiation in media with NPs are indicated (*p* < 0.05).

Analysis of the obtained results showed that the addition of colloidal solutions of AgNPs, FeNPs, MgNPs nanoparticles to the nutrient medium with *I. obliquus* inoculum stimulated the synthesis of intracellular melanins. The greatest effect was recorded when using AgNPs, FeNPs, the increase was 175% and 325%, respectively, compared to the control (without NPs) (Figure 9).

The synthesis of melanin pigments increases by 25–140% when exposed to low-intensity laser light and colloidal solutions of nanoparticles. The enhancing effects of laser light and nanoparticles can be ranked as follows: FeNPs < AgNPs < MgNPs. The nanoparticles studied stimulated the synthesis of melanin pigments, a non-enzymatic part of the fungal antioxidant system, and increased the level of antioxidant protection in *I. obliquus*, thereby increasing their stress resistance. It should be noted that the increase in melanin synthesis did not correlate with the accumulation of mycelial mass of fungi, for which a decrease in mycelial growth activity was recorded with photoinduction of all nanoparticles. The inhibitory effect of laser light and nanoparticles on growth activity can be ranked as follows: FeNPs (15.7 g/l) < AgNPs (14.7 g/l) < MgNPs (11.7 g/l).

## 4. Discussion

For the normal development and functioning of any biological system, including fungi, the presence of certain mineral substances necessary for growth and development is important. These substances can be presented in the form of mineral salts, components of organic macromolecules, chelate compounds, or in the form of nanoparticles of individual chemical elements [7]. Compared with conventional materials, nanoparticles have unique characteristics such as size, shape, modification, surface charge, hydrophobicity, which ensure their high bioavailability for living cells [40]. For example, the size of nanoparticles allows them to easily overcome biological barriers (cell membranes), and the diversity of the structure of nanoparticles can significantly increase the bioavailability and effectiveness of drugs, including for the agro-industrial complex [3,7–9]. As is known, nanoscale objects are characterized by a large (compared to macro-objects) ratio of surface area to volume (~1/d, where d is the characteristic size of the object), which radically increases the relative role of the surface, its properties and the influence of interaction with the environment on the surface of nanoparticles [41]. On the surface of nanoparticles, both chemical and physical processes occur, which are important for the interaction of nanoparticles with the cells of living organisms. Chemical interactions lead to the formation of new chemical compounds involving the nanoparticle material, as well as chemical catalysis processes, where the nanoparticle material does not participate in the reaction and is not consumed. Physical processes on the surface of nanoparticles result in the redistribution of electric charges on the surface, excitation of surface oscillations, particularly plasmons and surface excitons, changes in the physical structure of the surface, etc.

Depending on the reaction mechanism, nanoparticles can be divided into two groups: 1) nanoparticles, which quickly enter into chemical reactions and are a form of delivery of necessary substances to the body: microelements, vitamins, enzymes, drugs; 2) nanoparticles that are quite stable and retain their chemical composition, size, and shape for a long time, for example, nanoparticles of some metals, in particularly noble ones – gold, platinum, silver or carbon nanoparticles – nanotubes, fullerenes, graphene flakes [42,43]. Nanoparticles of the second group can remain in the body for a relatively long time, causing a catalytic effect, entering into physical interactions with cell membranes or other elements, and influencing biochemical processes in the body [44]. The presence of nanoparticles in a biological environment can affect its optical properties, which changes the conditions for light propagation and its interaction with the environment in such an environment.

Recently, a number of studies have been published on the use of photoactivated nanoparticles, indicating the great interest and potential importance of this problem [11,45,46]. Under certain conditions, the catalytic properties of materials can change when exposed to light, in particular, they can be enhanced. Li et al. (2022) proposed a method for synthesizing azetidine through photoinduced copper catalysis via radical cascade cyclization. This approach introduced a pharmacologically significant N-heterocyclic ring system into a complex molecular environment, allowing for the modification of drugs and derivatives of natural products [47].

Photocatalysis has also been established for nanoparticles, which act as catalysts in several biochemical reactions [11,48]. Photocatalysis involving nanoparticles is actively used in wastewater treatment technologies, agricultural technologies, and other areas [49–52]. However, works on this topic mainly describe the effects of biological activity, while the issues of their mechanisms remain largely open, although they are actively discussed in the literature [53]. This situation stimulates experiments with a number of variable parameters to search, on the one hand, for new methods of influencing the development of biological systems, and on the other hand, to expand the experimental database on possible mechanisms of biological activity of nanoparticles. Indeed, the stimulating effect of light of a certain spectral composition at the corresponding stages of development of mycelial fungal cultures has been confirmed by numerous experiments [11–13,33–35,54]. Several studies have shown that the presence of nanoparticles in a medium through which light passes causes a number of effects, such as excitation of local plasmons [55], formation of zones with increased light concentration around nanoparticles [56], light scattering by nanoparticles [57], changes in the fluorescent properties of molecules under the influence of nanoparticles [58], and others. Excitation of surface electromagnetic oscillations (plasmons) on the surface of nanoparticles by light changes the conditions of their interaction with biological systems, including cell membranes and the intercellular environment [45,46]. At the same time, the role of surface effects associated with the rate of chemical reactions and catalytic activity, factors important for the course of biological processes, increases significantly, and they can become dominant. It is also known that areas of local amplification of the light field (‘nano-jets’) are formed near metal nanoparticles [59], which can affect the photostimulation of biochemical processes, photodynamic effects in living systems, in particular, taking into account the mechanisms of action of low-intensity optical radiation on cells based on the presence of strong gradients of the light field. Such mechanisms are considered one of the probable factors determining the stimulating effect of low-intensity optical, in particular laser, radiation on the growth and development of plants and fungi. All these effects can potentially affect the interaction of biological molecules with light and change its effectiveness. On the other hand, one can also expect a reverse effect of light on the efficiency of interaction of nanoparticles with biological molecules, in particular due to the excitation of plasmonic oscillations on the surface of nanoparticles [60], and also through known mechanisms of photocatalytic activity [61]. All these factors provide reasonable grounds for conducting experiments in the format we propose.

In our experiment, we used nanoparticles of metals – silver, iron, and magnesium. Compounds of these metals form the basis of some drugs widely used in biology [51,62–67]. The main factor determining the effectiveness of NPs is their ability to interact with cells. The size of NPs plays a crucial role in their cellular interaction and also determines their biodistribution. The uptake of NPs in cells differs from the uptake of small molecules, and understanding these differences is critical for comprehension. A number of review and experimental studies have been published on the interaction of metal nanoparticles with plant and fungal cells [7,29,68–76].

It is known that the plasma membrane of a cell limits the penetration of large molecules and particles, allowing only small ones to pass through. It serves as the primary interface in the interaction between the cell and nanoparticles and acts as a barrier to their penetration. It defines the boundary and maintains the necessary intracellular environment of the cell. Small and nonpolar molecules, such as CO₂ and O₂, can easily diffuse through the lipid bilayer, but polar molecules, such as ions and large molecules, are unable to cross the plasma membrane on their own. The diameter of the pores (on average 5–20 nm) limits the size of nanoparticles capable of crossing the plasma membrane. However, there is evidence that nanoparticles can modulate the size of cell wall pores, thereby removing rigid structural constraints and reaching the plasmalemma [7]. In nature, important ions and proteins are transported across the lipid bilayer through specialized membrane transport protein channels. Upon contact with the cell membrane, most nanomolecules are internalized through endocytosis. A series of experimental studies on the mechanism of interaction of nanoparticles with cells of living organisms was conducted [7,77,78]. It has been established that the kinetics of uptake and saturation concentration of cells with nanoparticles vary depending on the size and shape of NPs [7,78]. The surface chemical properties of NPs play a crucial role in their interaction with cells. Positively charged NPs rapidly penetrate via clathrin-mediated endocytosis. A strong correlation has been established between the amount of positive charge and its internalization into the cell [7]. Thus, the factors influencing the interaction and uptake of NPs are related to the physicochemical properties of NPs and the biophysical properties of cell membranes [79].

Colloidal solutions of biogenic metal nanoparticles such as Ag, Cu, Fe, Mg, Mn, Mo, and Zn are currently being used in crop production at very low concentrations (10^−10^ M) to produce environmentally friendly products. Because of their nanosize, these particles can easily enter cells and affect plant growth and development. They also possess antibacterial and antioxidant properties by inducing internal protective mechanisms. Furthermore, these solutions optimize metabolic processes and help in the realization of the adaptive and productive potential of plants in varying weather and climatic conditions during their development. Additionally, research is being conducted on colloidal solutions of biologically active metals obtained through nanotechnology, which have anti-stress properties and enhance the resistance of plants, bacterias, and fungi cells to various adverse factors. Behra et al. (2013) studied the bioavailability of nanoparticles and silver ions from both chemical and biochemical standpoints [65].

Silver nanoparticles (AgNPs) exhibit enhanced catalytic activity due to their small size and high surface area. They are currently receiving significant attention in biomedicine for the treatment of various acute infectious diseases [80]. AgNPs are utilized as biosensors because of their optical properties and their ability to absorb and scatter light. Moreover, the bactericidal effect of silver nanoparticles is widely employed in dressings, antimicrobial coatings, and biomedical devices [80–83]. Among noble metal nanoparticles, AgNPs are the most chemically biocompatible. Despite their inertness, AgNPs effectively interact with antimicrobial compounds, leading to the production of reactive oxygen species such as hydrogen peroxide, which enhances antimicrobial activity. Recently, AgNPs have also been found to possess antiangiogenic, antipermeability, and anti-inflammatory potential, making them a valuable tool in the healthcare field [9].

The differences in the influence of the biogenic metal nanoparticles and laser irradiation we studied on the biosynthetic activity of *I. obliquus* can be explained by the difference in their ability to penetrate the fungal cells, different mechanisms of biochemical action, and optical properties. It is possible that they can act as signaling molecules because their nanomolar concentrations significantly change melanin synthesis. Analytical transmission microscopy revealed that FeNPs preferentially penetrate cells and can concentrate in vacuoles, whereas AgNPs are not detected in the intracellular space but are localized predominantly on the membrane surface [79,84].

Our results confirmed the previously established photo-stimulating effect of low-intensity laser irradiation on the growth and biosynthetic activity of various macromycetes, including *I. obliquus* [35,36,85,86]. Furthermore, it was found that photoinduced metal nanoparticles can significantly influence the growth processes (mycelial mass synthesis) and biosynthetic activity (synthesis of polysaccharides, flavonoids, and melanins) of *I. obliquus*. The use of photoinduced nanoparticles demonstrated both stimulating and inhibiting properties. For example, when the inoculum of *I. obliquus* was irradiated with laser light in a medium containing NPs, the growth activity of the mycelium decreased by 12.3% (for the medium containing FeNPs) to 35.4% (for the medium containing MgNPs). At the same time, a strong stimulating effect on the synthesis of IPS was observed when the inoculum of *I. obliquus* was irradiated in a medium with AgNPs. The differences in the influence of the metal nanoparticles and laser irradiation on the biosynthetic activity of *I. obliquus* can be explained by their varying abilities to penetrate fungal cells, different biochemical mechanisms of action, and optical properties.

According to the literature, *I. obliquus* synthesizes several phenolic components such as small phenolic compounds, flavonoids, polyphenols, melanins, and hydrolyzable tannins [16,87]. All chaga phenolic compounds have been shown to have high free-radical scavenging ability [16,88,89]. Studies *in vitro* and *in vivo* have shown that phenolic compounds can reduce the incidence of diseases caused by oxidative stress, including cancer, hypertension, neurodegenerative diseases, and autoimmune diseases [16,90]. Therefore, these compounds may have potential for pharmaceutical application. The greatest photo-stimulating effect on the accumulation of intracellular flavonoids was observed when photoinduced silver nanoparticles (AgNPs) were added to the medium. The minimal effect when exposed to laser light and nanoparticles was recorded for extracellular flavonoids.

Melanins belong to the group of high-molecular organic pigments formed during the oxidative polymerization of indole and phenolic compounds. They contain unpaired electrons in the form of stable free radicals, which can further react with metal ions or some proteins. It is the most common pigment among known biopigments and is widely present in animal, plant, and fungal cells. Melanins protect fungal organisms from unfavorable environmental conditions (ultraviolet light, extreme temperatures, oxidizing agents, heavy metals, radionuclides, hydrolytic enzymes) [91]. They act as an extracellular redox buffer that can neutralize oxidants produced by environmental stress. The protective effect is due to the ability of melanin pigments, through a reversible process of oxidation and reduction, to remove free radicals (reactive forms of nitrogen and oxygen) that are formed in response to stress factors, and to stabilize the level of redox potential in cells. Due to their physicochemical properties (thermo- and photostability), fungal melanins exhibit a wide range of biological properties: antioxidant, genoprotective, sorption, and photoprotective properties. Thus, melanins play a significant role in the formation of stress resistance and adaptation of fungal organisms to various environmental factors [91]. Melanins are easily synthesized by many types of fungi, so edible and medicinal macromycetes are considered today as promising producers of edible melanin, which can find important application as a drug against the effects of radiation. Natural melanins are nontoxic and effective when used [91,92]. In addition to protecting against radiation, melanins have semiconducting properties that can be used in the development of environmentally friendly electronic devices. Thus, there is a need to identify sources of melanin from edible biomaterials in fields as diverse as radioprotection and electronic design. Today, melanin has a wide range of industrial applications in medicine, food, cosmetics, and other fields [93,94].

According to the literature [79,84] nanoparticles can act as signaling molecules, as their nanomolar concentrations significantly alter melanin synthesis. Analytical transmission microscopy showed that FeNPs predominantly penetrate cells and can concentrate in vacuoles, whereas AgNPs are not found in the intracellular space but are mainly localized on the membrane surface [79,84]. Since the melanins of *I. obliquus* are primarily located in the cell wall, silver nanoparticles (AgNPs) likely serve as an inducer of melanin pigment synthesis. The positive response to the addition of AgNPs, where the stimulation of melanin synthesis exceeds the activation of growth processes, may be associated with forming a biologically active AgNP-melanin complex. This may be due to the high electron-acceptor capacity of melanin pigments, the presence of stable free radicals in high concentrations, and pronounced semiconductor properties. There is evidence in the literature indicating the potential formation of nanocomposites of melanin pigments and AgNPs in this fungus [9]. Additionally, due to the unique optical properties of AgNPs, laser irradiation may trigger mechanisms that inhibit melanin synthesis. At the same time, for FeNPs and MgNPs, laser irradiation stimulated melanin synthesis. However, there may be another explanation for the negative correlation between growth and melanin accumulation. This could be because cell metabolism is focused on cell division, leaving no additional resources for the biosynthesis and accumulation of certain chemical compounds in the cells

## 5. Conclusions

The photocatalytic activity of colloidal solutions of nanoparticles (AgNP, FeNP, and MgNP) obtained by electrospark processing of conductive materials as possible growth regulators and activators of biosynthetic activity of the biotechnologically important fungus *I. obliquus* was investigated. Significant differences in the growth and accumulation of biologically active compounds (polysaccharides, flavonoids, and melanins) were demonstrated when photoactivatable nanoparticles AgNPs, FeNP, and MgNP were added to the nutrient medium. Photoactivation of all the nanoparticles used with low-intensity laser light (λ = 488 nm) reduced the growth activity of *I. obliquus* by 12.3–35.4% compared to the control (without nanoparticles). At the same time, photoactivated AgNPs significantly intensified the synthesis of intracellular polysaccharides in *I. obliquus*, while photoactivated MgNPs increased the amount of exopolysaccharides in the culture fluid. The greatest effect on the stimulation of flavonoid synthesis was observed for photoactivated AgNPs. It was found that photoactivated FeNPs and MgNPs stimulated the synthesis of melanins. The greatest effect of photostimulation of melanin synthesis was observed with the addition of photoactivated iron nanoparticles. In light of the results obtained in the current study, the next steps will be related to optimizing the process of cultivating *I. obliquus in vitro* to further study the processes of photoreception in macromycetes and accumulate experimental results for innovative applications

## Research highlights

- Nanoparticles of metals boosted *I. obliquus* growth.
- Irradiation of the inoculum with laser light in a medium with nanoparticles reduced the growth activity of mycelium.
- Laser irradiation affected the synthesis of polysaccharides.
- Laser irradiation caused an intensification of the synthesis of flavonoids and melanin compounds in the mycelial mass.

## Supplementary Material

Graphical Abstract.jpg

Supplemental material_2 clean.docx

Supplemental material_1.docx

## Data Availability

The authors confirm that the data supporting the findings of this study are available within the article and its supplementary materials, and may be shared upon request
