# Supplementary figures and images for: Photoregulation of the biosynthetic activity of fungus Inonotus obliquus using colloidal solutions of biogenic metal nanoparticles and low-intensity laser radiation
Source: Bioengineered. 2025 Jan 28;16(1):2458371. doi: 10.1080/21655979.2025.2458371 (PMC11776471; doi:10.1080/21655979.2025.2458371)

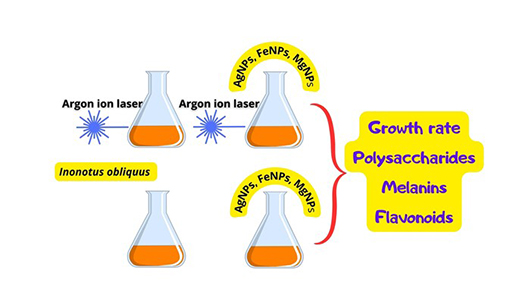

Supplement: Graphical Abstract.jpg [file KBIE_A_2458371_SM4643.jpg]
