# Supplementary material for: Photoregulation of the biosynthetic activity of fungus Inonotus obliquus using colloidal solutions of biogenic metal nanoparticles and low-intensity laser radiation
Source: Bioengineered. 2025 Jan 28;16(1):2458371. doi: 10.1080/21655979.2025.2458371 (PMC11776471; doi:10.1080/21655979.2025.2458371)
Supplement: Supplemental material_2 clean.docx [file KBIE_A_2458371_SM4642.docx]

**List of supplementary data**

**Supplementary Data S2**.

The implementation of the nanoparticle production method was carried out on a semi-industrial technological complex, which includes a discharge pulse generator, discharge chambers, and a control unit for the electrophysical parameters of the technological process (Figure 2)


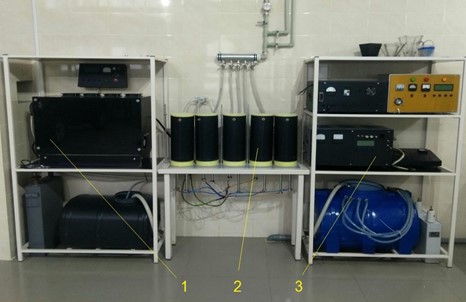
.

**Figure 2.** General view of the experimental setup for obtaining colloidal solutions of biogenic nanometals: 1 ‒ electro-spark discharge pulse generator; 2 ‒ discharge chambers; 3 ‒ control unit for electrophysical parameters of the technological process.

Our method for obtaining metal nanoparticles is based on the physical phenomenon of spark erosion of conductive materials. It is described in sufficient detail in the author's works:

1. Lopatko K.G., Olishevskyi V.V., Vinarchuk K.V., Lopatko S.K., Poedynok N.L., Babko E.M. Device for obtaining colloidal solutions of metals [Patent]. UA Patent 153591, July 6, 2023. Bulletin No. 30. National University of Bioresources and Nature Management of Ukraine [in Ukrainian]

2. Boretskij V.F., Veklich A.N., Tmenova T.A., Cressault Y., Valensi F., Lopatko S.K., Aftandilyants Y.G. Regulation of Biological Processes with Complexions of Metals Produced by Underwater Spark Discharge. In: Fesenko O., Yatsenko L. (eds) Nanooptics and Photonics, Nanochemistry and Nanobiotechnology, and Their Applications. Springer Proceedings in Physics, vol 247. Springer, Cham.(2020) <https://doi.org/10.1007/978-3-030-52268-1_23>.

3. Sergiienko . R., Ilkiv B., Petrovska S., Lopatko K., Lopatko S., Vinarchuk K., Hayasaka Yu., Tomai T., Verkhovliuk A., Zaulychnyy Yu. Structure and properties of silicon nano- and microparticles obtained by electric-spark dispersion method. Molecular Crystals and Liquid Crystals. 752(1), pp. 112-127. <https://doi.org/10.1080/15421406.2022.2091278>

4. Zakharchenko S.M., Shydlovska N.A., Perekos A.O., Lopatko K.G., Savluk O.S. Features of obtaining of plasma-erosion nanodispersed silver hydrosols and their bactericidal and fungicidal properties. Metallofizika i Noveishie Tekhnologii 42(6), 2020. pp. 829-851. <https://doi.org/10.15407/mfint.42.06.0829>

5. Veklich, A.., V. Boretskij, K. Lopatko, et. all. Hydrogen Balmer spectral lines in spectroscopy of underwater electric spark discharge plasma. Contributions of the Astronomical Observatory Skalnaté Pleso, vol.50, no. 1, pp. 96-104. <https://doi.org/10.31577/caosp.2020.50.1.96>

To obtain metal colloids, we used chemically pure starting materials (99.95% of the content of the corresponding metal) and deionized water with an electrical conductivity of no more than 20-30 microSiemens (μS). No other chemicals were present.

**Supplementary Data S3**.

In our research, we used an argon gas laser to generate coherent visible light at a wavelength of 488.0 nm. The laser intensity was regulated using an optical digital intensity and energy meter (PM-100D, Thorlabs Inc.) equipped with a standard photodiode voltage sensor S120C operating in the range of 400–1100 nm.


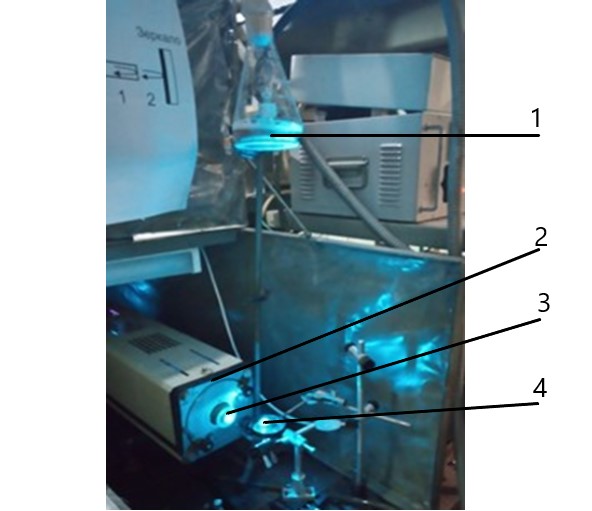


**Figure 3.** Laser installation for inoculum irradiation: 1 ‒ flask with mycelium; 2 – argon laser LGN-106M1; 3 – lens for shaping the illumination area; 4 – rotating mirror of the laser beam.
