# Supplementary material for: Photoregulation of the biosynthetic activity of fungus Inonotus obliquus using colloidal solutions of biogenic metal nanoparticles and low-intensity laser radiation
Source: Bioengineered. 2025 Jan 28;16(1):2458371. doi: 10.1080/21655979.2025.2458371 (PMC11776471; doi:10.1080/21655979.2025.2458371)
Supplement: Supplemental material_1.docx [file KBIE_A_2458371_SM4641.docx]

**List of supplementary data**

**Supplementary Data S1**. The *Inonotus obliquus* strain had macro- and micromorphological characteristics characteristic of this species, which can be used to monitor the purity of the culture *in vitro* constantly.

Figure 1. shows the morphology of mycelial colonies in various agar nutrient media. *I. obliquus* forms dense fluffy felt colonies on the media, zonate, at first white, becoming straw yellow, rarely cinnamon buff or buckthorn brown color.


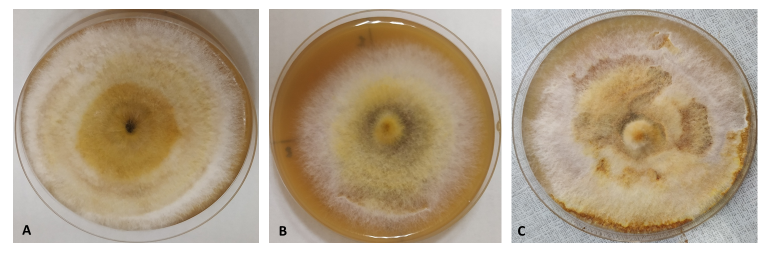
**Figure 1.** Morphology of mycelial colonies of *Inonotus obliquus* IBK 1877 on various nutrient agar media on the 14^th^ day of cultivation at a temperature of 26±1 °C: A – mycelial colony on a glucose-peptone-yeast agar (GPYA); B – mycelial colony on the malt extract agar (MEA); C – mycelial colony on the potato dextrose agar (PDA)

Micromorphological features of mycelium of .*I obliquus* IBK 1877


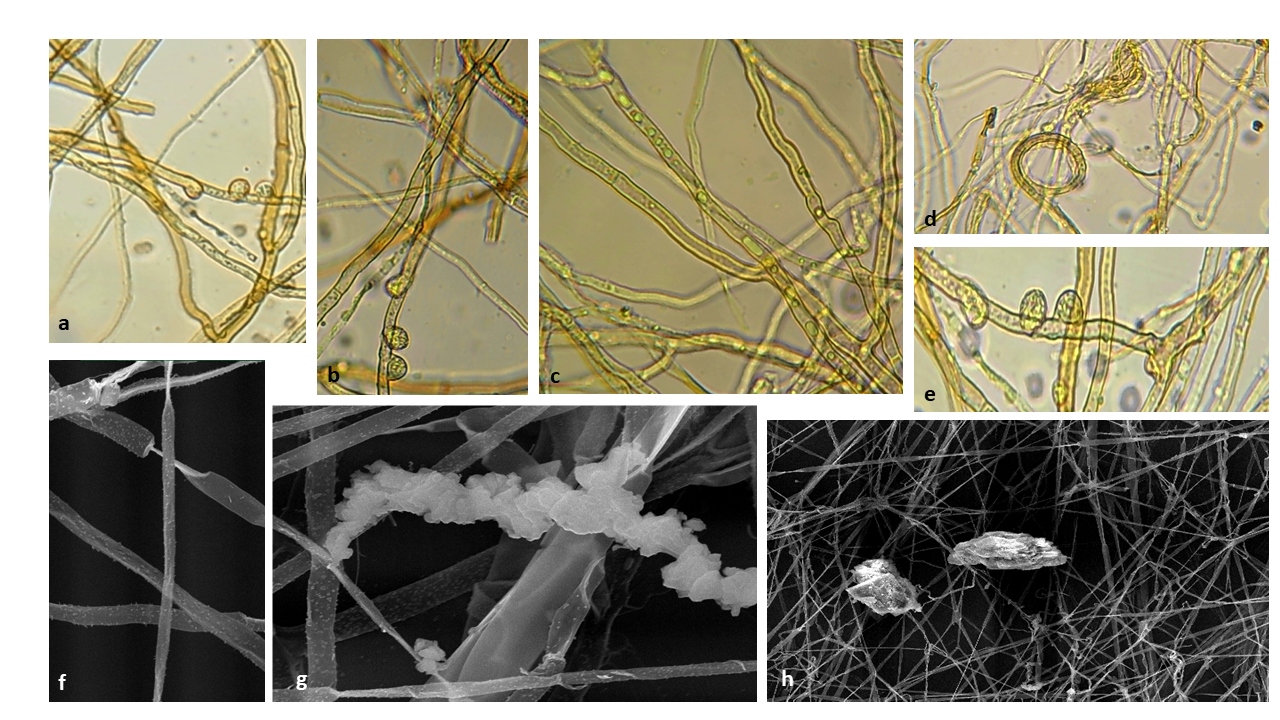


**Figure 2.** Micromorphological features of mycelium of *Inonotus obliquus* IBK 1877: hyphae and clamps (a, b, e), c ‒hyphae; d ‒ hyphal loops, light microscopy obj. × 40; f ‒ incrustated hyphae, SEM (× 3200), g ‒ mycelial mat of *Inonotus obliquus* with exometabolites SEM (× 200); C‒ exometabolites and incrustated hyphae, SEM (× 2600). Aerial hyphae are often incrustated and aggregation of exometabolites are present.

**Supplementary Data S2**.

The implementation of the nanoparticle production method was carried out on a semi-industrial technological complex, which includes a discharge pulse generator, discharge chambers, and a control unit for the electrophysical parameters of the technological process (Figure 2)


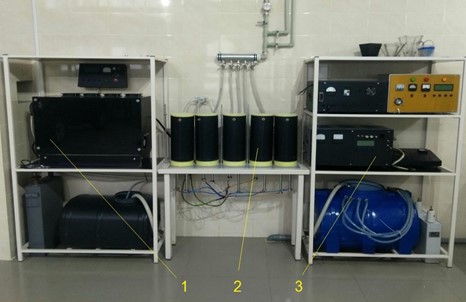
.

**Figure 2.** General view of the experimental setup for obtaining colloidal solutions of biogenic nanometals: 1 ‒ electro-spark discharge pulse generator; 2 ‒ discharge chambers; 3 ‒ control unit for electrophysical parameters of the technological process.

**Supplementary Data S3**.

In our research, we used an argon gas laser to generate coherent visible light at a wavelength of 488.0 nm. The laser intensity was regulated using an optical digital intensity and energy meter (PM-100D, Thorlabs Inc.) equipped with a standard photodiode voltage sensor S120C operating in the range of 400–1100 nm.


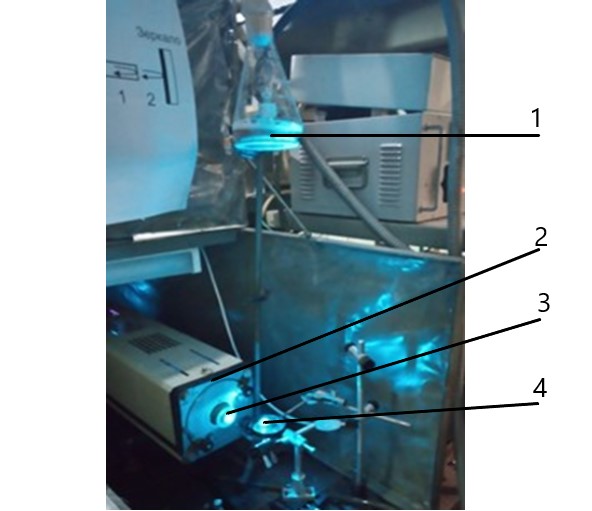


**Figure 3.** Laser installation for inoculum irradiation: 1 ‒ flask with mycelium; 2 – argon laser LGN-106M1; 3 – lens for shaping the illumination area; 4 – rotating mirror of the laser beam.
